# Supplementary material for: Role of Testing Conditions in Formation of Tribological Layers at Line Contacts of Antifriction CF-Reinforced PI- and PEI-Based Composites
Source: Molecules. 2022 Sep 27;27(19):6376. doi: 10.3390/molecules27196376 (PMC9571671; doi:10.3390/molecules27196376)
Supplement: Supplementary file 1 [file molecules-27-06376-s001.zip › molecules-1914597-SI.pdf]

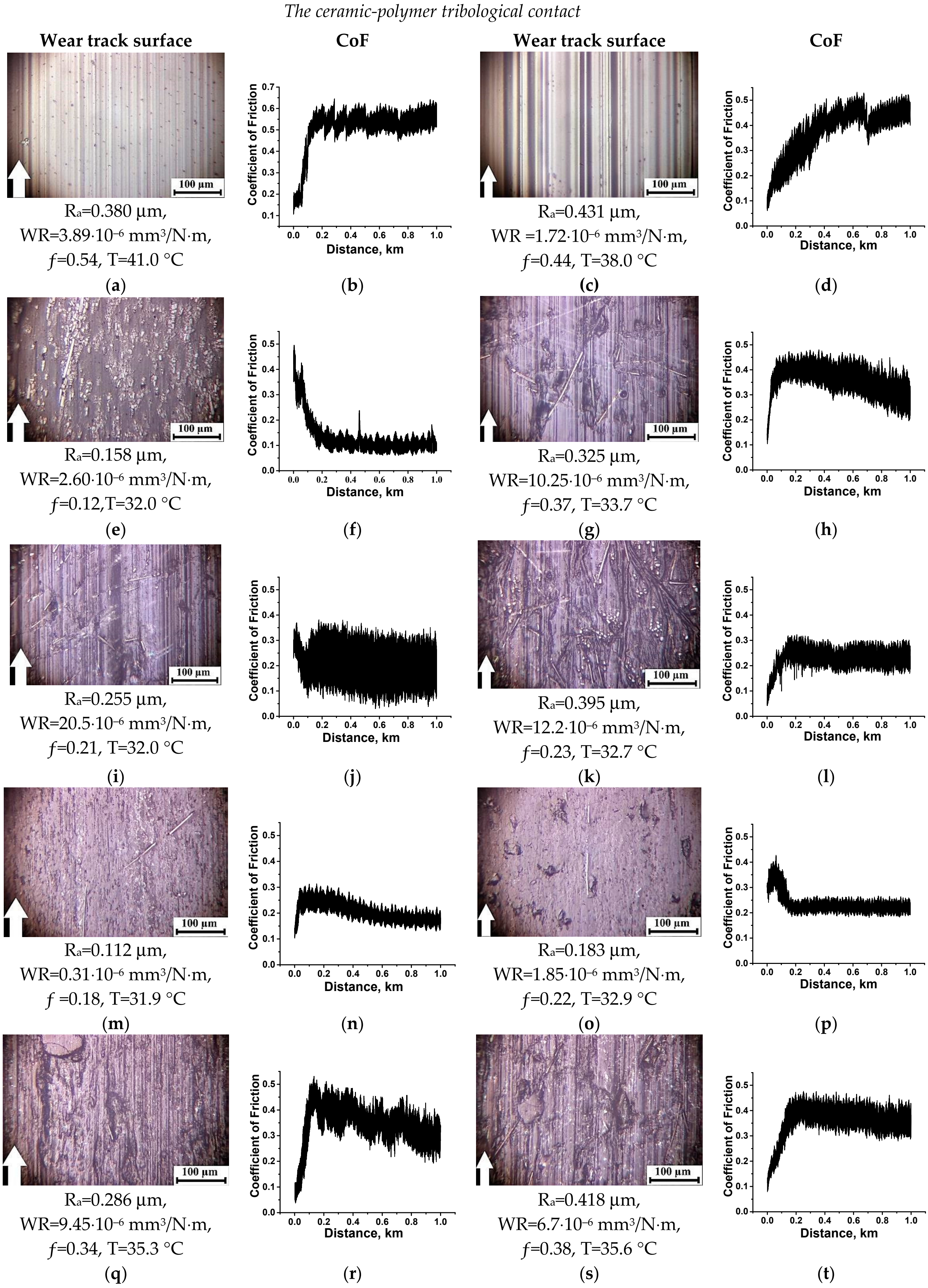
The ceramic-polymer tribological contact
Wear track surface
CoF
Wear track surface
CoF
100 µm
Coefficient of Friction
Distance, km
Ra=0.380 µm,
WR=3.89·10⁻⁶ mm³/N·m,
f=0.54, T=41.0 °C
(a)
(b)
Ra=0.431 µm,
WR =1.72·10⁻⁶ mm³/N·m,
f=0.44, T=38.0 °C
(c)
(d)
Ra=0.158 µm,
WR=2.60·10⁻⁶ mm³/N·m,
f=0.12,T=32.0 °C
(e)
(f)
Ra=0.325 µm,
WR=10.25·10⁻⁶ mm³/N·m,
f=0.37, T=33.7 °C
(g)
(h)
Ra=0.255 µm,
WR=20.5·10⁻⁶ mm³/N·m,
f=0.21, T=32.0 °C
(i)
(j)
Ra=0.395 µm,
WR=12.2·10⁻⁶ mm³/N·m,
f=0.23, T=32.7 °C
(k)
(l)
Ra=0.112 µm,
WR=0.31·10⁻⁶ mm³/N·m,
f =0.18, T=31.9 °C
(m)
(n)
Ra=0.183 µm,
WR=1.85·10⁻⁶ mm³/N·m,
f=0.22, T=32.9 °C
(o)
(p)
Ra=0.286 µm,
WR=9.45·10⁻⁶ mm³/N·m,
f=0.34, T=35.3 °C
(q)
(r)
Ra=0.418 µm,
WR=6.7·10⁻⁶ mm³/N·m,
f=0.38, T=35.6 °C
(s)
(t)

Figure S1. The optical micrographs of the wear track surfaces and the CoF time dependences for neat both PI (**a**, **b**) and PEI (**c**, **d**), as well as their PI/10CCF (**e**, **f**), PI/10CCF/10PTFE (**i**, **j**), PI/10CCF/10Gr (**m**, **n**), PI/10CCF/10MoS$_2$ (**q**, **r**), PEI/10CCF (**g**, **h**), PEI/10CCF/10PTFE (**k**, **l**), PEI/10CCF/10Gr (**o**, **p**), and PEI/10CCF/10MoS$_2$ (**s**, **t**) composites. The ceramic-polymer tribological contact under the 'mild' conditions (*P*=60 N, *V*=0.1 m/s).

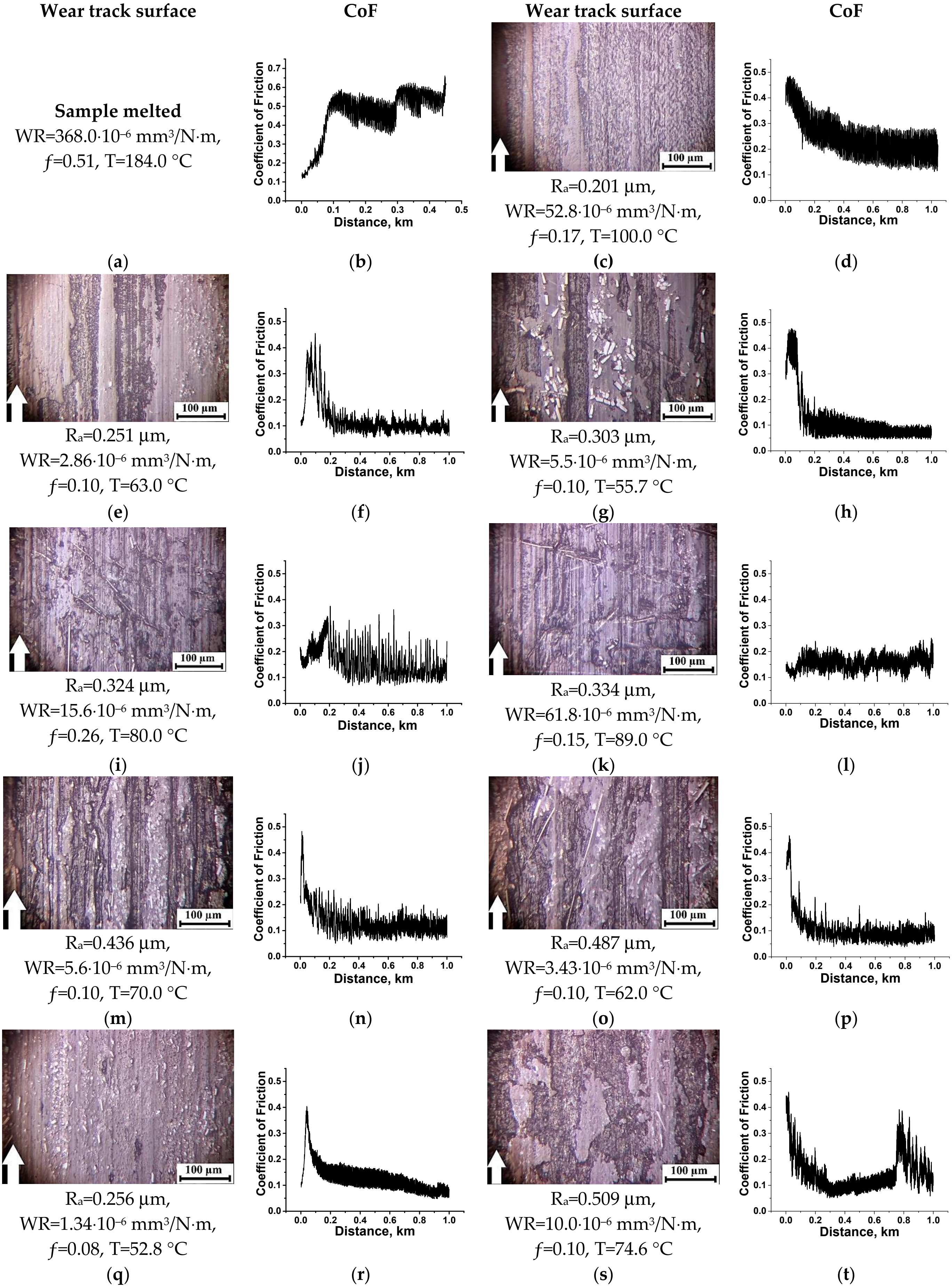

Wear track surface
CoF
Wear track surface
CoF
Sample melted
WR=368.0·10^{-6} mm^3/N·m,
f=0.51, T=184.0 °C
(a)
Coefficient of Friction
Distance, km
(b)
100 µm
R_a=0.201 µm,
WR=52.8·10^{-6} mm^3/N·m,
f=0.17, T=100.0 °C
(c)
(d)
R_a=0.251 µm,
WR=2.86·10^{-6} mm^3/N·m,
f=0.10, T=63.0 °C
(e)
(f)
R_a=0.303 µm,
WR=5.5·10^{-6} mm^3/N·m,
f=0.10, T=55.7 °C
(g)
(h)
R_a=0.324 µm,
WR=15.6·10^{-6} mm^3/N·m,
f=0.26, T=80.0 °C
(i)
(j)
R_a=0.334 µm,
WR=61.8·10^{-6} mm^3/N·m,
f=0.15, T=89.0 °C
(k)
(l)
R_a=0.436 µm,
WR=5.6·10^{-6} mm^3/N·m,
f=0.10, T=70.0 °C
(m)
(n)
R_a=0.487 µm,
WR=3.43·10^{-6} mm^3/N·m,
f=0.10, T=62.0 °C
(o)
(p)
R_a=0.256 µm,
WR=1.34·10^{-6} mm^3/N·m,
f=0.08, T=52.8 °C
(q)
(r)
R_a=0.509 µm,
WR=10.0·10^{-6} mm^3/N·m,
f=0.10, T=74.6 °C
(s)
(t)

Figure S2. The optical micrographs of the wear track surfaces and the CoF time dependences for neat both PI (**a**, **b**) and PEI (**c**, **d**), as well as their PI/10CCF (**e**, **f**), PI/10CCF/10PTFE (**i**, **j**), PI/10CCF/10Gr (**m**, **n**), PI/10CCF/10MoS$_2$ (**q**, **r**), PEI/10CCF (**g**, **h**), PEI/10CCF/10PTFE (**k**, **l**), PEI/10CCF/10Gr (**o**, **p**), and PEI/10CCF/10MoS$_2$ (**s**, **t**) composites. The ceramic-polymer tribological contact under the 'severe' conditions (P=180 N, V=0.5 m/s).

The metal-polymer tribological contact

**Wear track surface**	**CoF**	**Wear track surface**	**CoF**

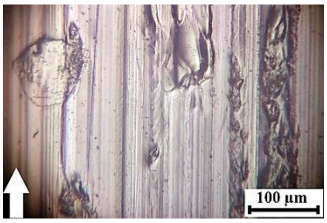

R_a=0.389 μm,
WR=33.6·10^{-6} mm^3/N·m,
f=0.50, T=28.6 °C

(**a**)

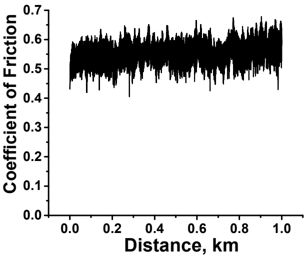

(**b**)

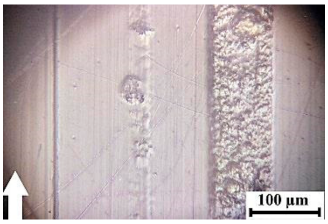

R_a=0.098 μm,
WR =7.71·10^{-6} mm^3/N·m,
f=0.41, T=26.6 °C

(**c**)

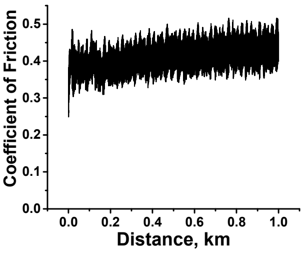

(**d**)

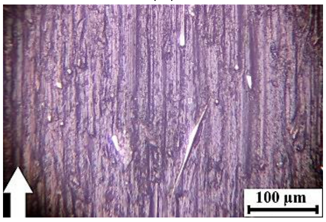

R_a=0.505 μm,
WR=2.5·10^{-6} mm^3/N·m,
f=0.26, T=26.0 °C

(**e**)

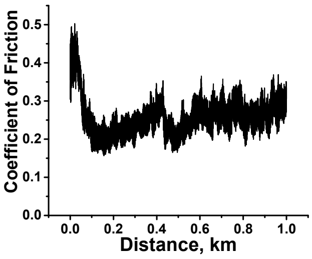

(**f**)

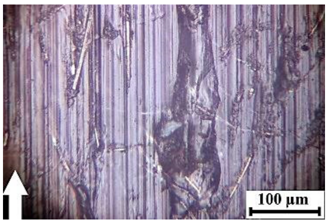

R_a=0.446 μm,
WR=13.6·10^{-6} mm^3/N·m,
f=0.35, T=27.8 °C

(**g**)

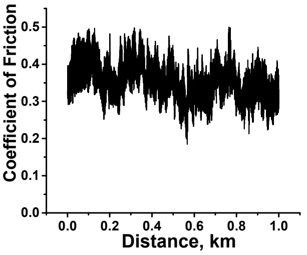

(**h**)

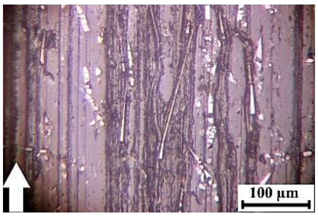

R_a=0.446 μm,
WR=31.5·10^{-6} mm^3/N·m,
f=0.28, T=24.6 °C

(**i**)

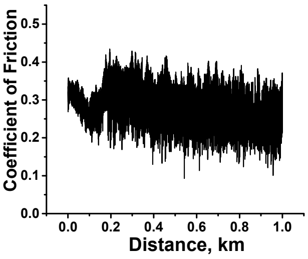

(**j**)

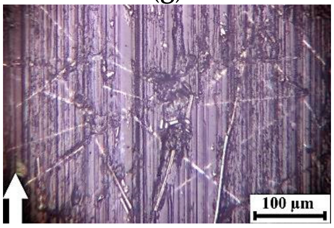

R_a=0.352 μm,
WR=13.4·10^{-6} mm^3/N·m,
f=0.22, T=25.9 °C

(**k**)

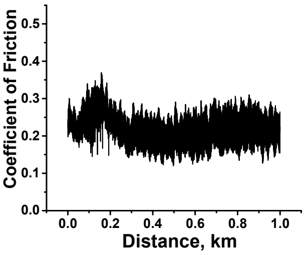

(**l**)

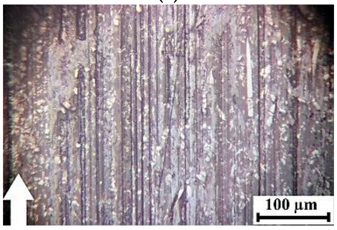

R_a=0.270 μm,
WR=1.86·10^{-6} mm^3/N·m,
f=0.25, T=26.9 °C

(**m**)

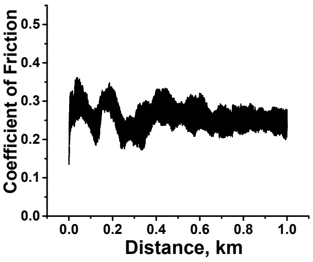

(**n**)

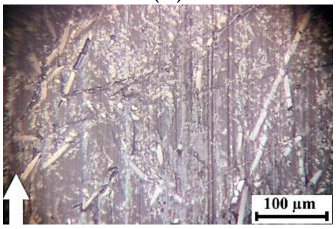

R_a=0.260 μm,
WR=1.7·10^{-6} mm^3/N·m,
f=0.23, T=24.9 °C

(**o**)

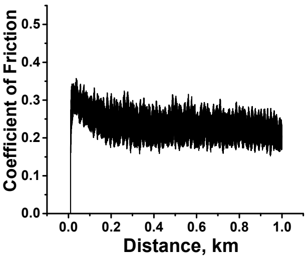

(**p**)

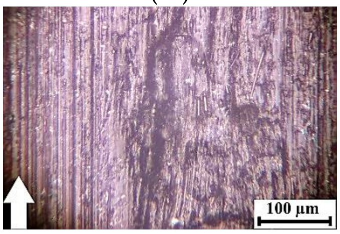

R_a=0.556 μm,
WR=4.32·10^{-6} mm^3/N·m,
f=0.38, T=23.7 °C

(**q**)

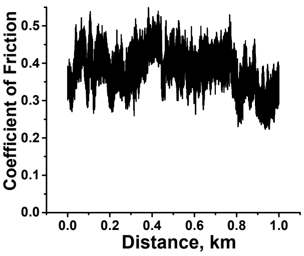

(**r**)

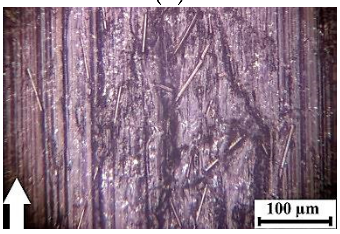

R_a=0.382 μm,
WR=6.64·10^{-6} mm^3/N·m,
f=0.33, T=27.2 °C

(**s**)

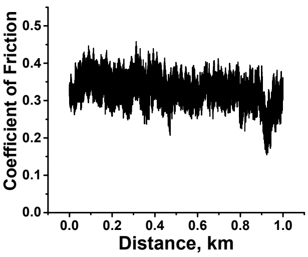

(**t**)

Figure S3. The optical micrographs of the wear track surfaces and the CoF time dependences for neat both PI (**a**, **b**) and PEI (**c**, **d**), as well as their PI/10CCF (**e**, **f**), PI/10CCF/10PTFE (**i**, **j**), PI/10CCF/10Gr (**m**, **n**), PI/10CCF/10MoS$_2$ (**q**, **r**), PEI/10CCF (**g**, **h**), PEI/10CCF/10PTFE (**k**, **l**), PEI/10CCF/10Gr (**o**, **p**), and PEI/10CCF/10MoS$_2$ (**s**, **t**) composites. The metal-polymer tribological contact under the 'mild' conditions (P=60 N, V=0.1 m/s).

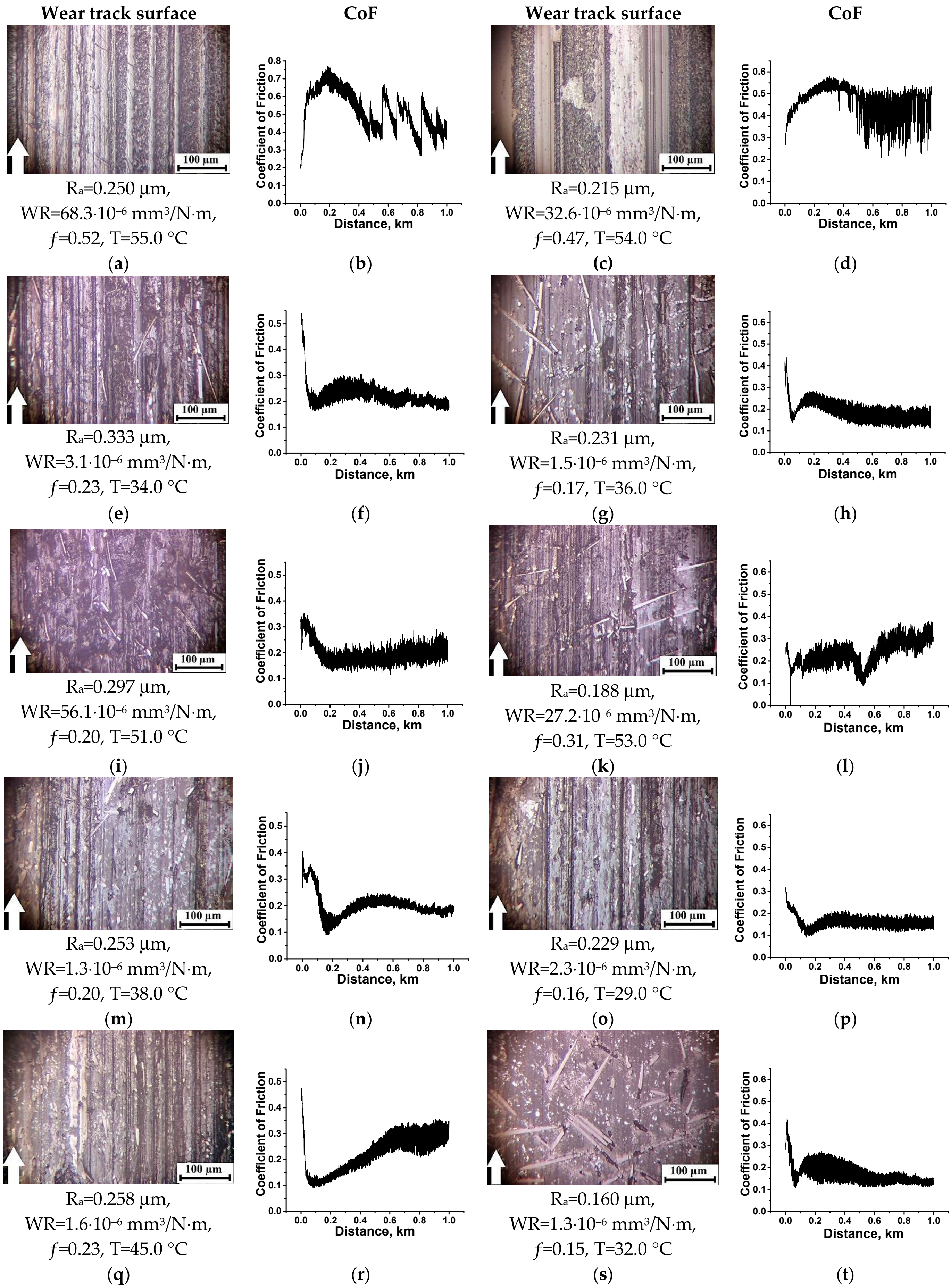
Wear track surface
CoF
Wear track surface
CoF
100 μm
Coefficient of Friction
Distance, km
Ra=0.250 μm, WR=68.3·10−6 mm3/N·m, f=0.52, T=55.0 °C
(a)
(b)
Ra=0.215 μm, WR=32.6·10−6 mm3/N·m, f=0.47, T=54.0 °C
(c)
(d)
Ra=0.333 μm, WR=3.1·10−6 mm3/N·m, f=0.23, T=34.0 °C
(e)
(f)
Ra=0.231 μm, WR=1.5·10−6 mm3/N·m, f=0.17, T=36.0 °C
(g)
(h)
Ra=0.297 μm, WR=56.1·10−6 mm3/N·m, f=0.20, T=51.0 °C
(i)
(j)
Ra=0.188 μm, WR=27.2·10−6 mm3/N·m, f=0.31, T=53.0 °C
(k)
(l)
Ra=0.253 μm, WR=1.3·10−6 mm3/N·m, f=0.20, T=38.0 °C
(m)
(n)
Ra=0.229 μm, WR=2.3·10−6 mm3/N·m, f=0.16, T=29.0 °C
(o)
(p)
Ra=0.258 μm, WR=1.6·10−6 mm3/N·m, f=0.23, T=45.0 °C
(q)
(r)
Ra=0.160 μm, WR=1.3·10−6 mm3/N·m, f=0.15, T=32.0 °C
(s)
(t)

Figure S4. The optical micrographs of the wear track surfaces and the CoF time dependences for neat both PI (**a**, **b**) and PEI (**c**, **d**), as well as their PI/10CCF (**e**, **f**), PI/10CCF/10PTFE (**i**, **j**), PI/10CCF/10Gr (**m**, **n**), PI/10CCF/10MoS_2 (**q**, **r**), PEI/10CCF (**g**, **h**), PEI/10CCF/10PTFE (**k**, **l**), PEI/10CCF/10Gr (**o**, **p**), and PEI/10CCF/10MoS_2 (**s**, **t**) composites. The metal-polymer tribological contact under the 'severe' conditions (P=180 N, V=0.5 m/s).

Table S1. The results of EDS analysis on the wear track surfaces shown in Figure 8, a–c.

Element	Spectrum 1 at. %	Spectrum 2 at. %	Spectrum 3 at. %	Spectrum 4 at. %	Spectrum 5 at. %
			PI		
C	70.99	70.38	62.48	66.42	70.60
O	26.09	26.74	33.69	29.96	26.27
S	2.81	2.79	3.43	1.41	2.80
Fe	0.10	0.09	0.39	2.21	0.33
			PI/10CCF		
C	5.08	70.00	13.22	71.05	21.65
O	59.48	26.63	46.06	25.32	49.40
S	2.43	3.27	1.92	3.48	1.58
Fe	33.00	0.10	38.80	0.15	27.37
			PI/10CCF/10Gr		
C	0.46	13.991	73.74	15.47	6.06
O	61.39	58.44	23.02	31.02	59.47
S	2.08	3.09	0.94	4.22	3.12
Fe	36.07	24.57	2.30	49.28	31.36

Table S2. The results of EDS analysis on the wear track surfaces shown in Figure 8, d–f.

Element	Spectrum 1 at. %	Spectrum 2 at. %	Spectrum 3 at. %	Spectrum 4 at. %	Spectrum 5 at. %
			PEI		
C	63.40	63.48	61.47	62.39	64.17
O	36.60	36.52	38.53	37.61	35.83
			PEI/10CCF		
C	34.73	87.16	17.81	20.23	46.19
O	38.82	12.39	52.60	53.80	36.50
Fe	26.46	0.45	29.60	25.97	17.30
			PEI/10CCF/10Gr		
C	9.85	21.42	74.22	1.78	7.47
O	58.96	50.67	25.61	61.44	63.10
Fe	31.20	27.91	0.17	36.78	29.43

Table S3. The results of EDS analysis on the wear track surfaces shown in Figure 9, a–c.

Element	Spectrum 1 at. %	Spectrum 2 at. %	Spectrum 3 at. %	Spectrum 4 at. %	Spectrum 5 at. %
			PI		
C	65.51	71.60	68.77	68.82	67.87
O	29.86	25.63	27.92	27.79	28.86
S	4.63	2.77	3.31	3.40	3.28
			PI/10CCF		
C	69.43	63.53	91.44	70.23	71.14
O	27.75	31.90	7.68	26.26	24.79
S	2.82	4.56	0.89	3.51	4.06
			PI/10CCF/10Gr		

C	60.38	45.65	62.43	78.04	72.30
O	38.12	47.99	32.82	17.54	25.43
S	1.34	2.44	3.45	4.30	2.27

Table S4. The results of EDS analysis on the wear track surfaces shown in Figure 9, d–f.

Element	Spectrum 1 at. %	Spectrum 2 at. %	Spectrum 3 at. %	Spectrum 4 at. %	Spectrum 5 at. %
PEI					
C	76.09	71.63	73.35	70.78	72.66
O	23.91	28.37	26.65	29.22	27.34
PEI/10CCF					
C	76.48	74.29	76.21	76.58	73.94
O	23.52	25.71	23.79	23.42	26.06
PEI/10CCF/10Gr					
C	76.37	84.66	82.02	77.73	73.89
O	23.63	15.34	17.98	22.27	26.11
